# Methyl 2-{6-[(1-meth­oxy-1-oxopropan-2-yl)amino­carbon­yl]pyridine-2-carboxamido}­propano­ate

**DOI:** 10.1107/S1600536812022258

**Published:** 2012-05-23

**Authors:** Mohamed A. Al-Omar, Abdel-Galil E. Amr, Hazem A. Ghabbour, Tze Shyang Chia, Hoong-Kun Fun

**Affiliations:** a Department of Pharmaceutical Chemistry, College of Pharmacy, King Saud University, Riyadh 11451, Saudi Arabia; b Drug Exploration & Development Chair (DEDC), College of Pharmacy, King Saud University, Riyadh 11451, Saudi Arabia; c Applied Organic Chemistry Department, National Research Center, Dokki 12622, Cairo, Egypt; d X-ray Crystallography Unit, School of Physics, Universiti Sains Malaysia, 11800 USM, Penang, Malaysia

## Abstract

In the title compound, C_15_H_19_N_3_O_6_, the amide planes are inclined at dihedral angles of 0.8 (6) and 12.1 (3)° with respect to the central pyridine ring. The mean planes of the corresponding methyl acetate groups form dihedral angles of 41.76 (13) and 86.48 (15)°, respectively with the mean plane of pyridine ring. A pair of weak intra­molecular N—H⋯N hydrogen bonds generate an *S*(5)*S*(5) ring motif in the mol­ecule. In the crystal, mol­ecules are linked by N—H⋯O hydrogen bonds into [001] chains. The chains are cross-linked by C—H⋯O hydrogen bonds into layers lying parallel to *bc* plane. The crystal packing also features a C—H⋯π inter­action.

## Related literature

For the synthesis and biological activity screening of some dipicolinic acid bis-l-amino acid hydrazide derivatives and their corresponding acids, see: Abou-Ghalia & Amr (2004 ▶); Al-Salahi *et al.* (2010 ▶); Al-Omar & Amr (2010 ▶); Attia *et al.* (2000 ▶). For the biological activity of 2,6-disubstituted pyridine derivatives, see: Amr (2005 ▶); Abou-Ghalia *et al.* (2003 ▶); Amr, Sayed & Abdulla (2005 ▶); Amr *et al.* (2006 ▶); Hammam *et al.* (2003 ▶). For hydrogen-bond motifs, see: Bernstein *et al.* (1995 ▶).
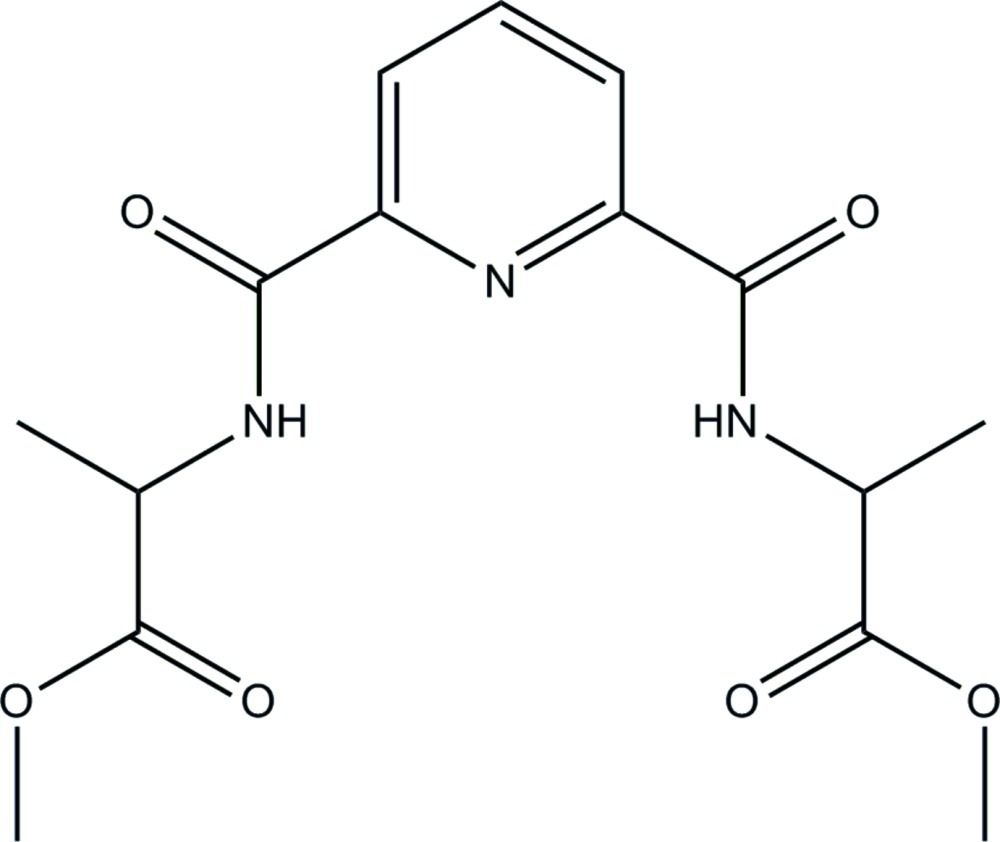


## Experimental

### 

#### Crystal data

C_15_H_19_N_3_O_6_
*M* *_r_* = 337.33
Monoclinic, 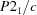

*a* = 8.9735 (3) Å
*b* = 20.7073 (8) Å
*c* = 10.4048 (5) Å
β = 122.901 (3)°
*V* = 1623.29 (11) Å^3^
*Z* = 4
Cu *K*α radiation
μ = 0.91 mm^−1^
*T* = 296 K
0.74 × 0.25 × 0.06 mm

#### Data collection

Bruker SMART APEXII CCD diffractometer
Absorption correction: multi-scan (*SADABS*; Bruker, 2009 ▶) *T*_min_ = 0.551, *T*_max_ = 0.947
10358 measured reflections
2703 independent reflections
2058 reflections with *I* > 2σ(*I*)
*R*_int_ = 0.046

#### Refinement

*R*[*F* ^2^ > 2σ(*F* ^2^)] = 0.053
*wR*(*F* ^2^) = 0.192
*S* = 1.04
2703 reflections
229 parameters
H atoms treated by a mixture of independent and constrained refinement
Δρ_max_ = 0.25 e Å^−3^
Δρ_min_ = −0.28 e Å^−3^

### 

Data collection: *APEX2* (Bruker, 2009 ▶); cell refinement: *SAINT* (Bruker, 2009 ▶); data reduction: *SAINT*; program(s) used to solve structure: *SHELXTL* (Sheldrick, 2008 ▶); program(s) used to refine structure: *SHELXTL*; molecular graphics: *SHELXTL*; software used to prepare material for publication: *SHELXTL* and *PLATON* (Spek, 2009 ▶).

## Supplementary Material

Crystal structure: contains datablock(s) global, I. DOI: 10.1107/S1600536812022258/hb6780sup1.cif

Structure factors: contains datablock(s) I. DOI: 10.1107/S1600536812022258/hb6780Isup2.hkl

Supplementary material file. DOI: 10.1107/S1600536812022258/hb6780Isup3.cml

Additional supplementary materials:  crystallographic information; 3D view; checkCIF report

## Figures and Tables

**Table 1:** Hydrogen-bond geometry (Å, °) *Cg*1 is the centroid of the N1/C1–C5 ring.

*D*—H⋯*A*	*D*—H	H⋯*A*	*D*⋯*A*	*D*—H⋯*A*
N3—H1*N*3⋯N1	0.85 (3)	2.23 (3)	2.676 (3)	113 (2)
N3—H1*N*3⋯O4^i^	0.85 (2)	2.35 (2)	3.080 (2)	145 (2)
N2—H1*N*2⋯N1	0.84 (3)	2.32 (3)	2.685 (3)	107 (3)
N2—H1*N*2⋯O4^i^	0.83 (3)	2.55 (3)	3.290 (3)	149 (2)
C9—H9*B*⋯O2^ii^	0.96	2.41	3.329 (3)	159
C15—H15*B*⋯*Cg*1^iii^	0.96	2.78	3.544 (4)	137

Symmetry codes: (i) 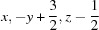; (ii) 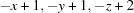; (iii) 
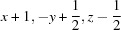
.

**Fig. 1.**
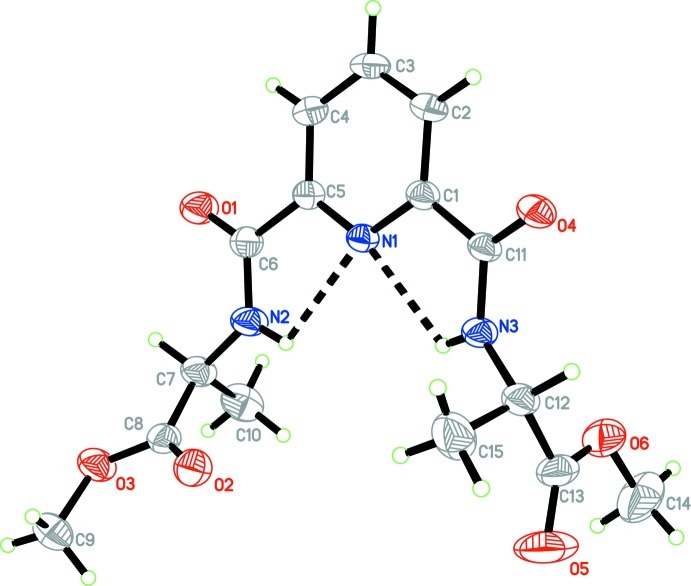
The molecular structure of the title compound with 30% probability displacement ellipsoids. The dashed lines represent the weak intramolecular N—H···N hydrogen bonds.

**Fig. 2.**
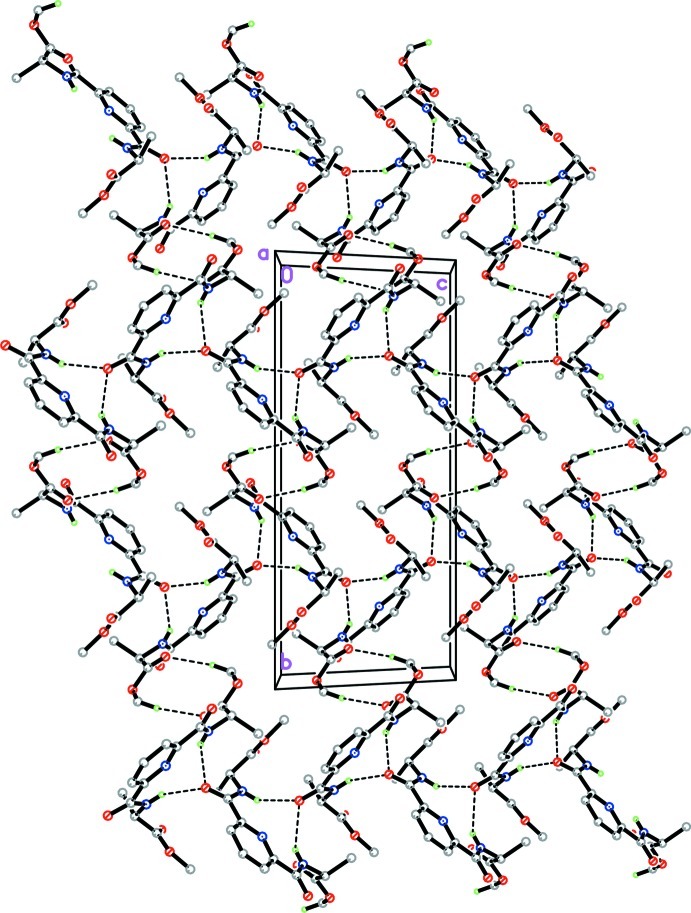
The crystal packing of the title compound. The dashed lines represent the hydrogen bonds. For clarity sake, hydrogen atoms not involved in hydrogen bonding have been omitted.

## supplementary crystallographic information

### Comment

In our previous work (Abou-Ghalia & Amr, 2004; Al-Salahi *et al.*, 2010; Al-Omar & Amr, 2010), we have reported the synthesis and biological activity screening of some dipicolinic acid bis-*L*-amino acid hydrazide derivatives and their corresponding acids (Attia *et al.*, 2000). In view of the significance of 2,6-disubstituted pyridine derivatives as biologically active congeners (Amr, 2005; Abou-Ghalia, Amr & Abdulla, 2003; Amr, Sayed & Abdulla, 2005; Amr *et al.*, 2006; Hammam *et al.*, 2003), we report herein the synthesis and crystal structure of the title compound.

The asymmetric unit of the title compound is shown in Fig. 1. The amide planes (O1/N2/C6 & O4/N3/C11) are inclined at dihedral angles of 0.8 (6) and 12.1 (3)°, respectively, with respect to the central pyridine ring (N1/C1–C5). The mean planes of the methyl acetate groups (O2/O3/C7–C9 with maximum deviation = 0.007 (2) Å at atom O3 & O5/O6/C12–C14 with maximum deviation = 0.011 (2) Å at atom O6) form dihedral angles of 41.76 (13) and 86.48 (15)°, respectively with the mean plane of pyridine ring. Weak intramolecular N2—H1N2···N1 and N3—H1N3···N1 hydrogen bonds (Table 1) generate an S(5)S(5) ring motif (Bernstein *et al.*, 1995) in the molecule.

In the crystal (Fig. 2), molecules are linked by intermolecular N3—H1N3···O4, N2—H1N2···O4 and C9—H9B···O2 hydrogen bonds (Table 1) into two-dimensional networks parallel to *bc* plane. The crystal packing is further stabilized by C—H···π interaction (Table 1), involving *Cg*1 which is the centroid of N1/C1–C5 ring.

### Experimental

To a cold mixture (-15 °C) of 2,6-pyridine dicarboxylic acid (0.167 g, 1 mmol) in cold dry tetrahydrofuran (100 ml) and ethyl chloroformate (0.216 g, 2 mmol), triethylamine (0.202 g, 2 mmol) was added with stirring. After 10 min, D-alanyl methyl ester (0.206 g, 2 mmol) was then added. The reaction mixture was stirred for 3 h at -15 °C and then 12 h at r.t. The triethylamine hydrochloride formed was filtered off and the solvent was evaporated under reduced pressure. The residue obtained was dissolved in 150 ml dichloromethane, washed with water, 1 N hydrochloric acid, 1 N sodium bicarbonate and finally with water and dried over anhydrous calcium chloride. The solvent was evaporated under reduced pressure to dryness and the obtained solid was crystallized from dichloromethane to give colourless plates of the title compound.

### Refinement

The atoms H1N2 and H1N3 were located in a difference fourier map and refined freely [N—H = 0.83 (3) and 0.85 (2) Å]. The remaining H atoms were positioned geometrically [C—H = 0.93, 0.96 and 0.98 Å] and refined using a riding model with *U*_iso_(H) = 1.2 or 1.5*U*_eq_(C). A rotating group model was applied to the methyl groups.

### Crystal data

**Table:**

C_15_H_19_N_3_O_6_	*F*(000) = 712
*M**_r_* = 337.33	*D*_x_ = 1.380 Mg m^−^^3^
Monoclinic, *P*2_1_/*c*	Cu *K*α radiation, λ = 1.54178 Å
Hall symbol: -P 2ybc	Cell parameters from 1828 reflections
*a* = 8.9735 (3) Å	θ = 5.5–70.4°
*b* = 20.7073 (8) Å	µ = 0.91 mm^−^^1^
*c* = 10.4048 (5) Å	*T* = 296 K
β = 122.901 (3)°	Plate, colourless
*V* = 1623.29 (11) Å^3^	0.74 × 0.25 × 0.06 mm
*Z* = 4	

### Data collection

**Table:**

Bruker SMART APEXII CCD diffractometer	2703 independent reflections
Radiation source: fine-focus sealed tube	2058 reflections with *I* > 2σ(*I*)
Graphite monochromator	*R*_int_ = 0.046
φ and ω scans	θ_max_ = 65.0°, θ_min_ = 5.5°
Absorption correction: multi-scan (*SADABS*; Bruker, 2009)	*h* = −10→10
*T*_min_ = 0.551, *T*_max_ = 0.947	*k* = −24→24
10358 measured reflections	*l* = −11→9

### Refinement

**Table:**

Refinement on *F*^2^	Primary atom site location: structure-invariant direct methods
Least-squares matrix: full	Secondary atom site location: difference Fourier map
*R*[*F*^2^ > 2σ(*F*^2^)] = 0.053	Hydrogen site location: inferred from neighbouring sites
*wR*(*F*^2^) = 0.192	H atoms treated by a mixture of independent and constrained refinement
*S* = 1.04	*w* = 1/[σ^2^(*F*_o_^2^) + (0.1344*P*)^2^] where *P* = (*F*_o_^2^ + 2*F*_c_^2^)/3
2703 reflections	(Δ/σ)_max_ < 0.001
229 parameters	Δρ_max_ = 0.25 e Å^−^^3^
0 restraints	Δρ_min_ = −0.28 e Å^−^^3^

### Special details

**Table:**

Geometry. All e.s.d.'s (except the e.s.d. in the dihedral angle between two l.s. planes) are estimated using the full covariance matrix. The cell e.s.d.'s are taken into account individually in the estimation of e.s.d.'s in distances, angles and torsion angles; correlations between e.s.d.'s in cell parameters are only used when they are defined by crystal symmetry. An approximate (isotropic) treatment of cell e.s.d.'s is used for estimating e.s.d.'s involving l.s. planes.
Refinement. Refinement of *F*^2^ against ALL reflections. The weighted *R*-factor *wR* and goodness of fit *S* are based on *F*^2^, conventional *R*-factors *R* are based on *F*, with *F* set to zero for negative *F*^2^. The threshold expression of *F*^2^ > σ(*F*^2^) is used only for calculating *R*-factors(gt) *etc*. and is not relevant to the choice of reflections for refinement. *R*-factors based on *F*^2^ are statistically about twice as large as those based on *F*, and *R*- factors based on ALL data will be even larger.

### Fractional atomic coordinates and isotropic or equivalent isotropic displacement parameters (Å^2^)

**Table:**

	*x*	*y*	*z*	*U*_iso_*/*U*_eq_	
O1	−0.1466 (3)	0.53103 (10)	0.8205 (2)	0.0711 (6)	
O2	0.4090 (3)	0.56823 (9)	0.89076 (19)	0.0641 (5)	
O3	0.2864 (3)	0.49181 (10)	0.7100 (2)	0.0710 (6)	
O4	0.2803 (2)	0.77285 (8)	1.37663 (16)	0.0578 (5)	
O5	0.6292 (4)	0.83396 (18)	1.1329 (3)	0.1239 (11)	
O6	0.3640 (4)	0.87058 (11)	1.0701 (2)	0.0823 (7)	
N1	0.0899 (2)	0.66141 (9)	1.07033 (18)	0.0438 (5)	
N2	0.0839 (3)	0.58945 (11)	0.8529 (2)	0.0602 (6)	
N3	0.3295 (3)	0.75674 (9)	1.1876 (2)	0.0497 (5)	
C1	0.0965 (3)	0.69752 (10)	1.1805 (2)	0.0449 (5)	
C2	−0.0180 (4)	0.68863 (13)	1.2288 (3)	0.0584 (7)	
H2A	−0.0101	0.7145	1.3054	0.070*	
C3	−0.1448 (4)	0.64055 (14)	1.1610 (3)	0.0638 (7)	
H3A	−0.2241	0.6337	1.1910	0.077*	
C4	−0.1521 (4)	0.60292 (13)	1.0484 (3)	0.0572 (6)	
H4A	−0.2358	0.5701	1.0016	0.069*	
C5	−0.0334 (3)	0.61483 (11)	1.0065 (2)	0.0471 (5)	
C6	−0.0374 (3)	0.57458 (12)	0.8840 (2)	0.0530 (6)	
C7	0.0902 (4)	0.55753 (13)	0.7325 (3)	0.0607 (7)	
H7A	0.0229	0.5172	0.7074	0.073*	
C8	0.2796 (4)	0.54102 (12)	0.7893 (3)	0.0550 (6)	
C9	0.4596 (4)	0.46969 (16)	0.7517 (3)	0.0766 (8)	
H9A	0.4468	0.4353	0.6845	0.115*	
H9B	0.5236	0.4543	0.8553	0.115*	
H9C	0.5236	0.5047	0.7430	0.115*	
C10	0.0058 (4)	0.59893 (17)	0.5880 (3)	0.0788 (9)	
H10A	−0.1141	0.6092	0.5555	0.118*	
H10B	0.0058	0.5755	0.5083	0.118*	
H10C	0.0726	0.6381	0.6093	0.118*	
C11	0.2444 (3)	0.74632 (10)	1.2579 (2)	0.0439 (5)	
C12	0.4982 (3)	0.79021 (13)	1.2598 (3)	0.0566 (6)	
H12A	0.5071	0.8179	1.3401	0.068*	
C13	0.5066 (5)	0.83318 (16)	1.1474 (3)	0.0723 (9)	
C14	0.3623 (7)	0.91492 (19)	0.9625 (4)	0.1168 (16)	
H14A	0.2584	0.9416	0.9187	0.175*	
H14B	0.3610	0.8911	0.8828	0.175*	
H14C	0.4665	0.9416	1.0144	0.175*	
C15	0.6504 (4)	0.74250 (17)	1.3363 (4)	0.0899 (10)	
H15A	0.6515	0.7215	1.4191	0.135*	
H15B	0.7605	0.7650	1.3753	0.135*	
H15C	0.6356	0.7107	1.2630	0.135*	
H1N3	0.289 (3)	0.7353 (11)	1.105 (3)	0.044 (6)*	
H1N2	0.151 (4)	0.6210 (14)	0.896 (3)	0.063 (8)*	

### Atomic displacement parameters (Å^2^)

**Table:**

	*U*^11^	*U*^22^	*U*^33^	*U*^12^	*U*^13^	*U*^23^
O1	0.0754 (13)	0.0729 (12)	0.0837 (11)	−0.0269 (10)	0.0553 (11)	−0.0275 (9)
O2	0.0620 (12)	0.0660 (11)	0.0641 (9)	−0.0096 (9)	0.0342 (9)	−0.0117 (8)
O3	0.0754 (14)	0.0750 (12)	0.0845 (12)	−0.0149 (10)	0.0576 (11)	−0.0296 (9)
O4	0.0738 (12)	0.0660 (10)	0.0528 (8)	−0.0078 (9)	0.0469 (9)	−0.0110 (7)
O5	0.115 (2)	0.176 (3)	0.136 (2)	−0.051 (2)	0.1048 (19)	−0.031 (2)
O6	0.1178 (18)	0.0758 (13)	0.0865 (12)	−0.0121 (13)	0.0770 (13)	0.0060 (10)
N1	0.0479 (11)	0.0482 (10)	0.0445 (9)	0.0006 (8)	0.0310 (9)	0.0015 (7)
N2	0.0646 (14)	0.0696 (14)	0.0651 (11)	−0.0223 (12)	0.0473 (11)	−0.0253 (10)
N3	0.0573 (13)	0.0580 (11)	0.0470 (9)	−0.0132 (9)	0.0369 (10)	−0.0131 (8)
C1	0.0506 (13)	0.0491 (12)	0.0440 (9)	0.0040 (10)	0.0315 (10)	0.0047 (8)
C2	0.0720 (17)	0.0656 (15)	0.0631 (12)	−0.0023 (13)	0.0533 (13)	−0.0044 (10)
C3	0.0662 (16)	0.0735 (17)	0.0811 (15)	−0.0105 (14)	0.0591 (14)	−0.0040 (12)
C4	0.0602 (16)	0.0566 (14)	0.0705 (14)	−0.0088 (12)	0.0457 (13)	−0.0010 (10)
C5	0.0490 (13)	0.0486 (12)	0.0503 (10)	0.0000 (10)	0.0312 (11)	0.0019 (9)
C6	0.0542 (15)	0.0541 (13)	0.0569 (11)	−0.0060 (11)	0.0343 (12)	−0.0062 (10)
C7	0.0639 (16)	0.0681 (16)	0.0653 (13)	−0.0184 (13)	0.0450 (13)	−0.0261 (11)
C8	0.0679 (17)	0.0549 (13)	0.0589 (12)	−0.0104 (13)	0.0452 (13)	−0.0090 (10)
C9	0.085 (2)	0.083 (2)	0.0836 (17)	0.0065 (17)	0.0600 (17)	−0.0092 (14)
C10	0.072 (2)	0.105 (2)	0.0569 (13)	−0.0048 (18)	0.0335 (15)	−0.0183 (14)
C11	0.0519 (13)	0.0489 (12)	0.0430 (10)	0.0039 (10)	0.0337 (10)	0.0022 (8)
C12	0.0583 (15)	0.0647 (15)	0.0617 (12)	−0.0148 (12)	0.0422 (12)	−0.0206 (10)
C13	0.086 (2)	0.084 (2)	0.0764 (16)	−0.0386 (18)	0.0634 (17)	−0.0317 (15)
C14	0.197 (5)	0.094 (2)	0.095 (2)	−0.044 (3)	0.103 (3)	−0.0047 (18)
C15	0.0597 (19)	0.085 (2)	0.122 (2)	−0.0032 (16)	0.0471 (18)	−0.0272 (19)

### Geometric parameters (Å, º)

**Table:**

O1—C6	1.227 (3)	C4—C5	1.375 (3)
O2—C8	1.204 (3)	C4—H4A	0.9300
O3—C8	1.334 (3)	C5—C6	1.507 (3)
O3—C9	1.442 (4)	C7—C8	1.504 (4)
O4—C11	1.225 (2)	C7—C10	1.527 (4)
O5—C13	1.189 (4)	C7—H7A	0.9800
O6—C13	1.329 (4)	C9—H9A	0.9600
O6—C14	1.441 (3)	C9—H9B	0.9600
N1—C5	1.340 (3)	C9—H9C	0.9600
N1—C1	1.343 (3)	C10—H10A	0.9600
N2—C6	1.329 (3)	C10—H10B	0.9600
N2—C7	1.445 (3)	C10—H10C	0.9600
N2—H1N2	0.83 (3)	C12—C13	1.504 (4)
N3—C11	1.331 (3)	C12—C15	1.514 (4)
N3—C12	1.449 (3)	C12—H12A	0.9800
N3—H1N3	0.85 (2)	C14—H14A	0.9600
C1—C2	1.379 (3)	C14—H14B	0.9600
C1—C11	1.507 (3)	C14—H14C	0.9600
C2—C3	1.383 (4)	C15—H15A	0.9600
C2—H2A	0.9300	C15—H15B	0.9600
C3—C4	1.378 (3)	C15—H15C	0.9600
C3—H3A	0.9300		
			
C8—O3—C9	117.4 (2)	O3—C9—H9A	109.5
C13—O6—C14	116.2 (3)	O3—C9—H9B	109.5
C5—N1—C1	117.75 (19)	H9A—C9—H9B	109.5
C6—N2—C7	121.9 (2)	O3—C9—H9C	109.5
C6—N2—H1N2	119 (2)	H9A—C9—H9C	109.5
C7—N2—H1N2	118 (2)	H9B—C9—H9C	109.5
C11—N3—C12	122.81 (17)	C7—C10—H10A	109.5
C11—N3—H1N3	114.2 (17)	C7—C10—H10B	109.5
C12—N3—H1N3	121.7 (17)	H10A—C10—H10B	109.5
N1—C1—C2	122.7 (2)	C7—C10—H10C	109.5
N1—C1—C11	116.56 (19)	H10A—C10—H10C	109.5
C2—C1—C11	120.61 (19)	H10B—C10—H10C	109.5
C1—C2—C3	118.7 (2)	O4—C11—N3	124.5 (2)
C1—C2—H2A	120.7	O4—C11—C1	120.8 (2)
C3—C2—H2A	120.7	N3—C11—C1	114.65 (17)
C4—C3—C2	119.1 (2)	N3—C12—C13	111.0 (2)
C4—C3—H3A	120.4	N3—C12—C15	110.5 (2)
C2—C3—H3A	120.4	C13—C12—C15	112.5 (3)
C5—C4—C3	118.7 (2)	N3—C12—H12A	107.5
C5—C4—H4A	120.6	C13—C12—H12A	107.5
C3—C4—H4A	120.6	C15—C12—H12A	107.5
N1—C5—C4	123.0 (2)	O5—C13—O6	124.6 (3)
N1—C5—C6	116.8 (2)	O5—C13—C12	123.2 (4)
C4—C5—C6	120.1 (2)	O6—C13—C12	112.2 (2)
O1—C6—N2	124.0 (2)	O6—C14—H14A	109.5
O1—C6—C5	120.5 (2)	O6—C14—H14B	109.5
N2—C6—C5	115.5 (2)	H14A—C14—H14B	109.5
N2—C7—C8	109.3 (2)	O6—C14—H14C	109.5
N2—C7—C10	111.4 (2)	H14A—C14—H14C	109.5
C8—C7—C10	111.4 (2)	H14B—C14—H14C	109.5
N2—C7—H7A	108.2	C12—C15—H15A	109.5
C8—C7—H7A	108.2	C12—C15—H15B	109.5
C10—C7—H7A	108.2	H15A—C15—H15B	109.5
O2—C8—O3	123.6 (2)	C12—C15—H15C	109.5
O2—C8—C7	125.8 (2)	H15A—C15—H15C	109.5
O3—C8—C7	110.6 (2)	H15B—C15—H15C	109.5
			
C5—N1—C1—C2	−0.4 (3)	C9—O3—C8—C7	−179.5 (2)
C5—N1—C1—C11	175.80 (18)	N2—C7—C8—O2	−25.0 (4)
N1—C1—C2—C3	0.0 (4)	C10—C7—C8—O2	98.6 (3)
C11—C1—C2—C3	−176.0 (2)	N2—C7—C8—O3	156.0 (2)
C1—C2—C3—C4	0.4 (4)	C10—C7—C8—O3	−80.5 (3)
C2—C3—C4—C5	−0.4 (4)	C12—N3—C11—O4	13.6 (4)
C1—N1—C5—C4	0.3 (3)	C12—N3—C11—C1	−165.8 (2)
C1—N1—C5—C6	−179.55 (19)	N1—C1—C11—O4	−166.69 (19)
C3—C4—C5—N1	0.1 (4)	C2—C1—C11—O4	9.5 (3)
C3—C4—C5—C6	179.9 (2)	N1—C1—C11—N3	12.8 (3)
C7—N2—C6—O1	4.1 (4)	C2—C1—C11—N3	−171.0 (2)
C7—N2—C6—C5	−176.5 (2)	C11—N3—C12—C13	−141.6 (2)
N1—C5—C6—O1	180.0 (2)	C11—N3—C12—C15	92.8 (3)
C4—C5—C6—O1	0.1 (4)	C14—O6—C13—O5	−0.3 (4)
N1—C5—C6—N2	0.5 (3)	C14—O6—C13—C12	178.2 (2)
C4—C5—C6—N2	−179.3 (2)	N3—C12—C13—O5	−131.9 (3)
C6—N2—C7—C8	−136.0 (3)	C15—C12—C13—O5	−7.5 (4)
C6—N2—C7—C10	100.4 (3)	N3—C12—C13—O6	49.5 (3)
C9—O3—C8—O2	1.4 (4)	C15—C12—C13—O6	173.9 (2)

### Hydrogen-bond geometry (Å, º)

*Cg*1 is the centroid of the N1/C1–C5 ring.

**Table:**

*D*—H···*A*	*D*—H	H···*A*	*D*···*A*	*D*—H···*A*
N3—H1*N*3···N1	0.85 (3)	2.23 (3)	2.676 (3)	113 (2)
N3—H1*N*3···O4^i^	0.85 (2)	2.35 (2)	3.080 (2)	145 (2)
N2—H1*N*2···N1	0.84 (3)	2.32 (3)	2.685 (3)	107 (3)
N2—H1*N*2···O4^i^	0.83 (3)	2.55 (3)	3.290 (3)	149 (2)
C9—H9*B*···O2^ii^	0.96	2.41	3.329 (3)	159
C15—H15*B*···*Cg*1^iii^	0.96	2.78	3.544 (4)	137

Symmetry codes: (i) *x*, −*y*+3/2, *z*−1/2; (ii) −*x*+1, −*y*+1, −*z*+2; (iii) *x*+1, −*y*+1/2, *z*−1/2.
